# Efficacy of the novel CDK7 inhibitor QS1189 in mantle cell lymphoma

**DOI:** 10.1038/s41598-019-43760-z

**Published:** 2019-05-10

**Authors:** Yun Jung Choi, Dong Ha Kim, Dok Hyun Yoon, Cheolwon Suh, Chang-Min Choi, Jae Cheol Lee, Jung Yong Hong, Jin Kyung Rho

**Affiliations:** 1 Asan Institute for Life Sciences, Asan Medical Center, University of Ulsan, College of Medicine, Seoul, 05505 South Korea; 2 Department of Oncology, Asan Medical Center, University of Ulsan, College of Medicine, Seoul, 05505 South Korea; 3 Department of Pulmonology and Critical Care Medicine, Asan Medical Center, University of Ulsan, College of Medicine, Seoul, 05505 South Korea; 4 Department of Convergence Medicine, Asan Medical Center, University of Ulsan, College of Medicine, Seoul, 05505 South Korea

**Keywords:** Targeted therapies, B-cell lymphoma

## Abstract

Mantle cell lymphoma (MCL) is typically an aggressive and rare form of non-Hodgkin lymphoma (NHL) with a poor prognosis despite recent advances in immunochemotherapy and targeted therapeutics against NHL. New therapeutic agents are needed for MCL. In this study, we generated a potent inhibitor of cyclin-dependent kinase 7 (CDK7), designated QS1189, and confirmed its anti-cancer effects towards MCL and other lymphomas. QS1189 was highly selective for CDK7 and showed potent anticancer effects in MCL compared to other targeted therapeutic agents, such as ibrutinib and venetoclax. Consistent with a conventional CDK7 inhibitor, QS1189 treatment significantly decreased phosphorylation of the carboxyl-terminal domain of RNA polymerase II and transcription-associated genes. QS1189 induced cell cycle arrest at the G2/M phase and apoptosis. Interestingly, QS1189 overcame the acquired resistance to venetoclax, which is mediated by Bcl-xL. Similarly, QS1189 showed potent tumour cell growth inhibition of various lymphomas. Thus, CDK7 might be a suitable therapeutic target for inhibiting lymphoma, and QS1189 is a promising therapeutic option for various lymphomas and cells with acquired resistance to targeted therapy.

## Introduction

Mantle cell lymphoma (MCL) is a relatively rare cancer affecting lymphoid cells and accounts for 3–10% of non-Hodgkin’s lymphoma cases in Western countries. In Korea, MCL accounts for approximately 2% of B-cell lymphomas. Despite the existence of an indolent subgroup (10–15% of MCL patients) that survives for more than 10 years, most cases exhibit an aggressive clinical course, short response to treatment, and median survival of only 3–5 years^1,2^. During the past decade, notable therapeutic advances have been achieved in MCL with high-dose cytarabine containing induction regimens, autologous stem cell transplantation, rituximab maintenance, and highly efficacious new agents (such as bendamustine, bortezomib, lenalidomide, ibrutinib and venetoclax). Despite these recent therapeutic advances and high response rate to initial therapy, most patients show continuous relapse and MCL remains an incurable disease^3–5^. Thus, new agents are urgently needed to improve disease management and patient survival.

Numerous recent studies of the molecular and cellular biology of MCL have provided new opportunities for mechanism-based therapy. Indeed, ibrutinib and venetoclax are two of the most widely used agents for treating MCL^6,7^. Bruton’s tyrosine kinase (BTK) is a mediator of B-cell receptor pathway activation associated with nuclear factor-κB pathway activation in diverse B-cell lymphomas. In patients with relapsed or refractory MCL, the selective BTK inhibitor ibrutinib showed substantial efficacy with the best overall response rate of 68%, complete response rate of 21%, and median progression-free survival of 13.9 months^7^. Additionally, dysregulation of apoptosis via overexpression of the antiapoptotic protein B-cell leukaemia/lymphoma-2 (Bcl-2) is a crucial step in several B-cell lymphomas. Venetoclax, a highly selective Bcl-2 inhibitor, showed the best overall response rate of 75%, complete response rate of 21%, and median progression-free survival of 14 months in a phase I first-in-human study in patients with relapsed or refractory MCL^8^. Although these two drugs exhibit substantial efficacy in patients with relapsed or refractory MCL, drug treatment is limited because of primary and acquired resistance. Many studies are currently underway to elucidate the mechanisms of primary and acquired resistance to ibrutinib or venetoclax and are concurrently exploring drug combinations to identify more effective therapeutics with longer disease-free survival.

Cyclin-dependent kinases (CDKs) are involved in controlling eukaryotic cell division and RNA polymerase II (RNAPII)-dependent transcription^9,10^. CDK7 is known to play a key role in the direct link between transcription regulation and the cell cycle, as a CDK-activating kinase that phosphorylates CDKs, and as an essential component of the transcription factor TFIIH^11^. CDK7 phosphorylates serine-5 in the C-terminal domain (CTD) repeat region of RNAPII and phosphorylates CDK9, which is responsible for phosphorylation of the RNAPII CTD at serine-2. These actions of CDK7 are essential for transcription by RNAPII^12^. Additionally, CDK7 modulates the activities of several transcription factors, including p53^13^, retinoid receptors^14,15^, androgen receptor^16,17^ and oestrogen receptor^18^. Treatment with CDK7 inhibitors reduced the gene expression and inhibited proliferation in cancer cell lines and animal models^19–21^. Previous studies demonstrated that the anticancer efficacy of CDK7 inhibitors was associated with CDK7-dependent transcriptional addiction in small cell lung cancer and ovarian cancer^20,22^ and super-enhancer-associated genes in neuroblastoma and oesophageal squamous cell carcinoma^19,23^. CDK7 inhibitors also significantly reduced the transcription of Myc, Mcl-1, and Bcl-xL which are important factors in the growth and survival of lymphoma^24,25^. Thus, the development of selective CDK7 inhibitors for cancer treatment has received widespread attention, and some CDK7 inhibitors currently are being examined in preclinical or clinical studies as cancer therapeutics^25–28^.

Here, we developed a new potent inhibitor targeting CDK7, QS1189, and investigated the efficacy and anticancer mechanisms this agent in MCL cells and various types of lymphoma cells.

## Results

### Development of novel CDK7 inhibitor

**Figure 1:**
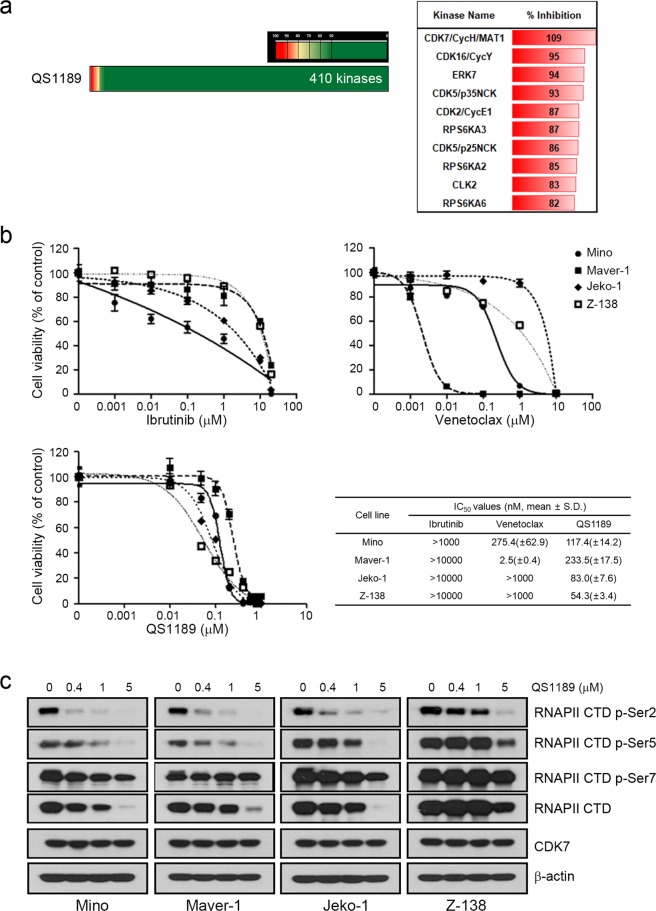
Characterization of the novel CDK7 inhibitor QS1189. (**a**) Selectivity and target profile of 1 μM QS1189 against approximately 410 kinases in a multikinase inhibition assay *in vitro*. Shown are kinases whose activity was inhibited by >80% at the dose tested. (**b**) MCL cells were treated with ibrutinib, venetoclax, and QS1189 for 72 h. Cell viability was evaluated using the MTT assay and IC_50_ values were calculated. (**c**) MCL ells were treated with the indicated dose of QS1189 for 6 h. The indicated protein levels were analysed by immunoblotting.

We generated a pharmaceutically active pyrazolo-triazine derivative as a selective inhibitor of CDK7. This compound was designated as QS1189. QS1189 potently inhibited CDK7 activity among a panel of 410 kinases in a multikinase inhibition assay *in vitro* (Fig. 1a and Supplemental Table S1). Although QS1189 showed similar inhibition to other CDKs including CDK16/CycY, CDK5/p35NCK, CDK2/CycE1, and CDK5/p25NCK, QS1189 inhibited CDK7 activity *in vitro* with an IC_50_ of 15 nM and showed IC_50_ values that were 10–600-fold higher than those of other CDKs (Supplemental Table S2).

The BTK inhibitor ibrutinib and Bcl-2 inhibitor venetoclax show antitumor activity in the treatment of MCL^6–8^. We tested the antitumor activity of QS1189 compared to those of conventional drugs such as ibrutinib and venetoclax in MCL cells. Most MCL cells showed resistance to ibrutinib and venetoclax, although venetoclax showed growth inhibition with an IC_50_ value in the nanomolar range (IC_50_ value of 275.4 ± 62.9 and 50, 2.5 ± 0.4 nM for Mino and Maver-1 cells, respectively; Fig. 1b). However, QS1189 inhibited the growth of MCL cells with IC_50_ values between 50 and 250 nM. Thus, QS1189 may be a more potent drug than other targeted agents for treating MCL.

### Preclinical efficacy of QS1189 in MCL

CDK7 kinase activity has been implicated in phosphorylation of the CTD of RNAPII which plays a role in transcription initiation and RNAPII procession^29–31^. To evaluate the dose-dependent inhibition of CDK7 substrates by QS1189, we performed immunoblotting following treatment with QS1189. QS1189 treatment completely inhibited the phosphorylation of CDK7 substrate RNAPII CTD at Ser-2 and Ser-5 and slightly reduced RNAPII p-Ser7 CTD phosphorylation (Fig. 1c). Interestingly, QS1189 treatment led to reduced RNAPII expression. These results were also observed after 24 h of QS1189 treatment (Supplemental Fig. S1).

**Figure 2:**
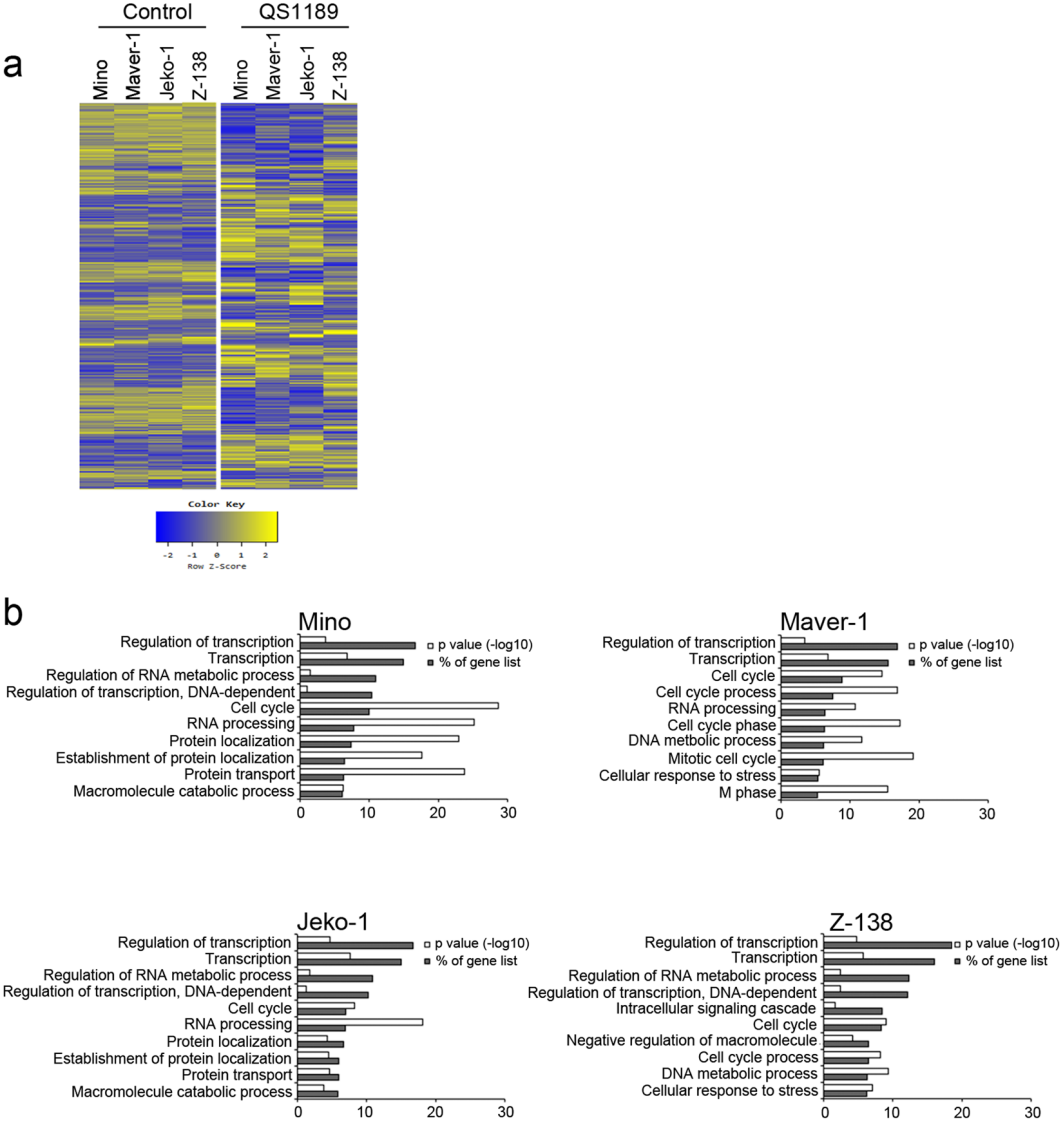
Transcriptional inhibition in QS1189 treated MCL cells. (**a**) Heatmap of gene expression in MCL cells treated with 1 μM QS1189 for 6 h. Rows show z-scores calculated for each cell. (**b**) GO analysis of 2-fold downregulated genes based on biological processes using DAVID software. The top 10 terms were ranked according to “% of gene list” that indicates the percentile of the gene count from the total number of genes in GO terms.

We examined the effect of CDK7 inhibition on gene expression in MCL cells following exposure to 1 µM QS1189 for 6 h by performing RNA sequencing. A total of 13,714 genes with detectable expression levels was retained, from which approximately 1,000 differentially expressed genes were downregulated in QS1189-treated MCL cells (Mino, 1793 genes; Maver-1, 1327 genes; Jeko-1, 1617 genes, Z-138, 1078 genes; log2-fold change ≤−2; Fig. 2a). Gene ontology analysis of downregulated genes revealed that genes involved in transcriptional regulation and cell cycle were enriched (Fig. 2b).

**Figure 3:**
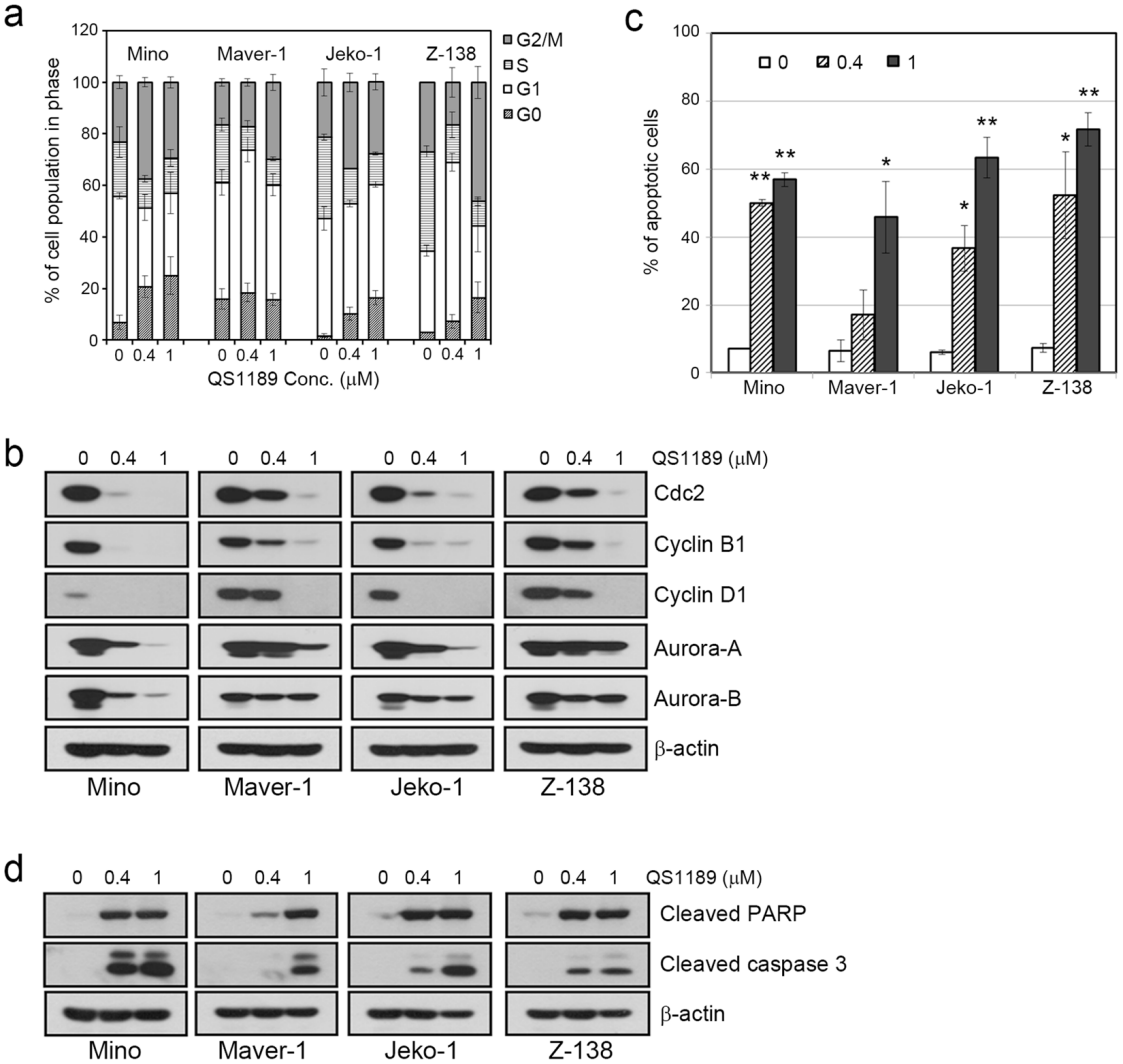
Induction of cell cycle arrest and apoptosis by QS1189 in MCL cells. (**a**) MCL cells were treated with QS1189 for 24 h prior to cell cycle analysis by propidium iodide (PI) staining. The experiment was repeated in triplicate. (**b**) G2/M related protein expression was detected by immunoblotting. (**c**) MCL cells were treated with QS1189 for 24 h, and then harvested and stained with Annexin V–FITC and PI. The percentage of apoptosis was the sum of the early apoptosis and late apoptosis population. Results are shown as the mean ± standard deviation of three independent experiments. *P < 0.05; **P < 0.005 compared to the control group. (**d**) Cleaved PARP and caspase-3 levels were detected by immunoblotting.

To better understand the mechanisms of the antitumor activity of QS1189 in MCL cells, we analysed apoptosis and the cell cycle. As shown in Fig. 2, QS1189 treatment induced G2/M arrest and apoptosis in all MCL cells (Fig. 3a,c). Consistent with these results, the expression of cdc2, cyclin B1, cyclin D1, and aurora-A/B, all of which participate in the G2/M phases, was significantly decreased (Supplemental Fig. S2), while QS1189 treatment induced apoptosis signalling, including PARP and caspase-3 activation (Fig. 3b,d). Taken together, these data suggest that QS1189 effectively inhibits the CDK7 substrate and induces G2/M arrest and apoptosis by suppressing transcription in MCL cells.

### Anticancer efficacy of QS1189 in cells with acquired resistance to venetoclax

**Figure 4:**
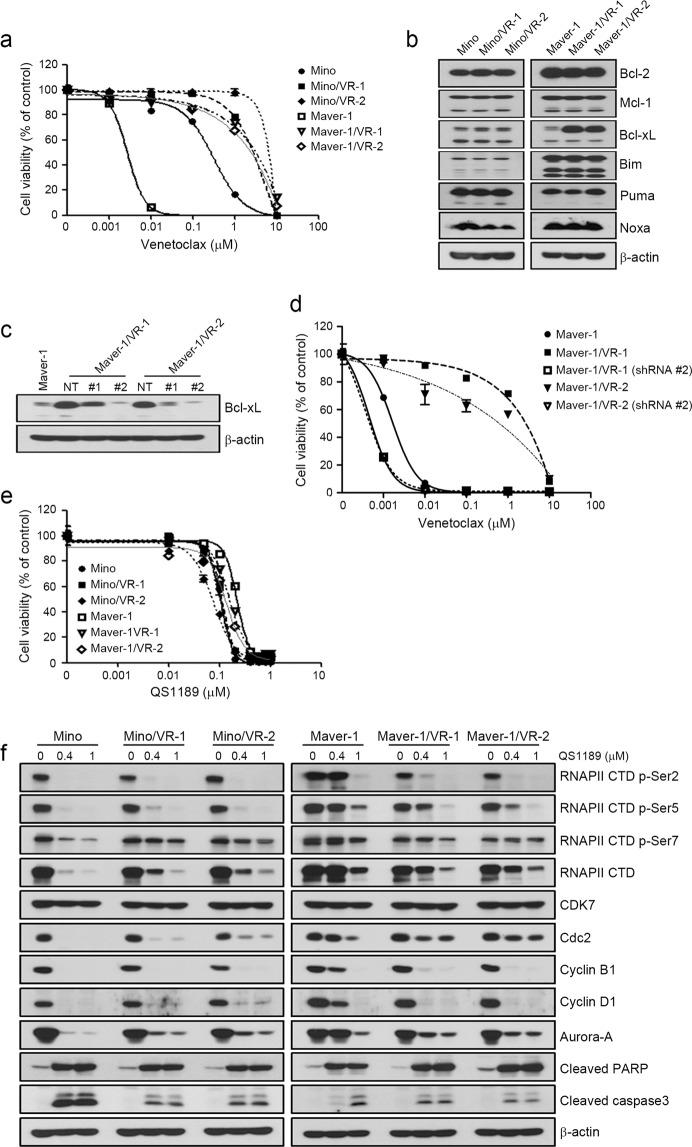
Efficacy of QS1189 in cells with acquired resistance to venetoclax. (**a**) Parental and venetoclax-resistant cells were treated with venetoclax at the indicated doses for 72 h. Cell viability was measured using the MTT assay. (**b**) Basal levels of pro- and anti-apoptosis bcl-2 family proteins in parental and venetoclax-resistant cells were analysed by immunoblotting. (**c**) Maver-1/VR-1 and Maver-1/VR-2 cells were stably transduced with lentivirus expressing shBcl-xL. Knockdown of Bcl-xL was confirmed by immunoblotting. (**d**) shBcl-xL-infected cells were treated with venetoclax for 72 h. Cell viability was assessed by MTT assay. (**e**) Cell viability was assessed by MTT analysis of parental and venetoclax-resistant cells treated with QS1189 for 72 h. (**f** ) Cells were treated with 1 μM QS1189 for 6 h. The indicated protein levels were analysed by immunoblotting.

To evaluate whether the acquisition of resistance to venetoclax affects the anticancer efficacy of QS1189, we established 2 venetoclax-resistant cell lines (Mino and Maver-1) by long-term venetoclax exposure. The sublines with acquired resistance to venetoclax included two clones (Mino/VR-1 and Mino/VR-2) from Mino and two clones (Maver-1/VR-1 and Maver-1/VR-2) from Maver-1 obtained by single-cell culture. As shown in Fig. 4a, all resistant cells showed an approximately 10–100-fold higher resistance to venetoclax than the parental cells, although Maver-1/VRs cells acquired higher resistance than Mino/VRs cells. Consistent with the results of a previous study^32^, the induction of Bcl-xL was observed in both Maver-1/VR-1 and Maver-1/VR-2 cells (Fig. 4b). To determine whether the induction of Bcl-xL was associated with acquired resistance to venetoclax, we suppressed the Bcl-xL gene using two different Bcl-xL-specific shRNAs; suppression of the Bcl-xL protein was confirmed by immunoblotting (Fig. 4c). The responsiveness to venetoclax was restored in resistant cells through silencing of Bcl-xL (Fig. 4d). Next, we examined whether the acquisition of resistance to venetoclax affected the antitumor effects of QS1189. Interestingly, the acquisition of resistance to venetoclax did not affect the sensitivity to QS1189 (Fig. 4e). In addition, QS1189 treatment showed reduction of Bcl-xL and Mcl-1 which were associated with acquired resistance to venotoclax (Supplemental Fig. S3). As observed in parental cells, CDK7 substrate inhibition, G2/M arrest, and apoptosis by QS1189 treatment were observed in all resistant cells (Fig. 4f). Although we did not determine the mechanisms of acquired resistance to venetoclax in Mino/VRs cells, QS1189 treatment overcame the acquired resistance to venetoclax.

### Preclinical efficacy of QS1189 in various lymphomas

**Figure 5:**
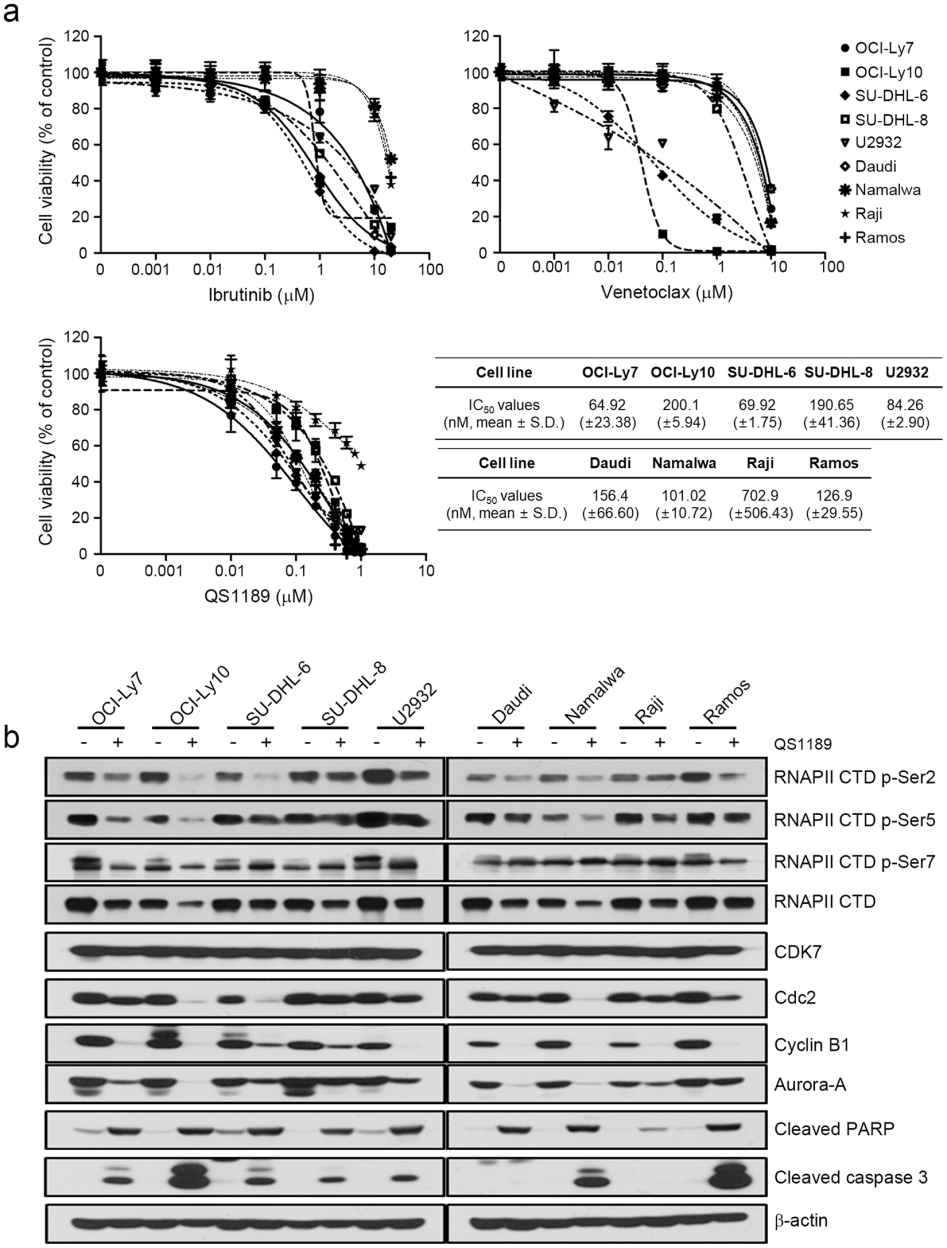
Effects of QS1189 in B cell lymphoma cells. (**a**) Cells were treated with ibrutinib, venetoclax, and QS1189 at the indicated doses for 72 h. Cell viability was determined using the MTT assay and IC_50_ values of QS1189 were calculated. (**b**) Cells were treated with the 1 μM QS1189 for 6 h. The indicated protein levels were analysed by immunoblotting.

We further examined whether the anticancer effects of QS1189 extend to other subtypes of lymphoma including 4 Burkitt’s lymphoma cell lines (Daudi, Namalwa, Raji, and Ramos) and 5 diffuse large B-cell lymphoma cell lines (OCI-Ly7, OCI-Ly10, SU-DHL-6, SU-DHL-8U2932 and U2932). The cells were treated with increasing concentrations of drugs for 72 h and growth inhibition was determined by the MTT assay (Fig. 5a). Although a few cell lines were sensitive to venetoclax (IC_50_ values of 35.34, 66.93, and 121.2 nM for OCI-LY10, SU-DHL6, and U2932 cells, respectively), most cell lines were resistant to ibrutinib and venetoclax. However, QS1189 treatment inhibited the growth of Burkitt’s lymphoma cells and diffuse large B-cell lymphoma cells with IC_50_ values between 1 and 100 nM except for Raji cells. Additionally, QS1189 inhibited the CDK7 substrate, with concurrent induction of G2/M arrest and apoptosis in all cell lines, although there was a slight difference between cell lines (Fig. 5b). Taken together, these data suggest that QS1189 has potent anticancer effects towards MCL cells and various lymphoma cells.

## Discussion

Treatment of mantle cell lymphoma is difficult because patients are typically diagnosed in stage IV, after the MCL has metastasised. Younger patients are typically treated with intensive chemotherapy or autologous stem cell transplantation^4,33,34^, whereas older patients are treated with tolerable regimens such as bendamustine and rituximab or R-CHOP with rituximab maintenance^35–37^. Although these regimens show a high response rate during initial therapy, most patients eventually relapse and succumb to their disease^3,4,33^. Thus, new effective drugs are needed.

CDK7 is considered as an attractive target for treating human cancer, as it controls the activity of key enzymes involved in cell cycle progression, including other cyclin-dependent kinases such as CDK1, CDK2, CDK4, and CDK6. CDK7 function regulates the cell cycle of metazoans by controlling CDK activation through T-loop phosphorylation^19^. Moreover, CDK7 represents a component of the general transcription factor TFIIH and facilitates efficient transcriptional initiation, pause release, and elongation by phosphorylation of serine residues of RNAPII CTD (C-terminal domain). In our study, CDK7 inhibition by QS1189 diminished bulk Ser2, Ser5, and Ser7 phosphorylation of the RNAPII CTD in MCL cells as well as other lymphoma cells. Interestingly, we found that CDK7 inhibition led to reduced RNAPII transcription, although the effects of RNAPII transcription by CDK7 are controversial. Along with these results, QS1189 treatment induced G2/M arrest by decreasing the levels of cyclin B1, Aurora, and Cdc2 and then apoptosis in all lymphoma cells. Thus, the anticancer mechanisms of QS1189 in lymphoma included the induction of G2/M arrest and apoptosis by reducing RNAPII activity and transcription.

CDK7 inhibition resulted in the reduction of cellular mRNA levels because of the inhibition of RNAPII-dependent transcription by affecting the stability of preinitiation complexes^38^. Some studies demonstrated that CDK7 inhibitors are potential therapeutic agents that suppress master transcription-regulating oncogenes and disrupt their attendant super-enhancers^19–21,39^. Importantly, QS1189 treatment showed strong transcriptional inhibition of cyclin D1, Mcl-1, and c-Myc. Cyclin D1 is the genetic hallmark of MCL, and c-Myc and Mcl-1 are potent oncogenes in large B-cell lymphomas. Aberrant expression of these factors was associated with tumour proliferation and clinical outcomes^40–42^. Although QS1189 led to global mRNA downregulation, these results provide rationale for lymphoma treatment.

Signalling pathways associated with the pathogenesis of lymphoma have revealed new therapeutic targets for clinical investigation including ubiquitin-proteasome signaling^43^, the Akt/mTOR pathway^44^, B-cell receptor signaling^45^, anti-apoptotic pathway^8^, and cell-mediated immunity^46^. The impressive efficacy of some targeted agents including ibrutinib and venetoclax has changed the standard for care in specific subsets of patients. However, the use of these targeted agents is limited because of the development of acquired resistance to these drugs. For example, the mechanisms of acquired resistance to venetoclax are involved in mutations in Bcl-2 family proteins^47^ and upregulation of Mcl-1 and Bcl-xL^48^. Consistent with the results of a previous study, we found that the upregulation of Bcl-xL was observed in cells with acquired resistance to venetoclax. Suppression of Bcl-xL led to the recovery of sensitivity to venentoclax in Maver-1/VR cells. However, the acquisition of resistance to venetoclax did not affect the sensitivity to QS1189. Although the mechanisms of acquired resistance to venetoclax in Mino/VR cells were not examined in the current study, QS1189 treatment may be an attractive treatment option for overcoming acquired resistance to venetoclax.

In conclusion, we developed the novel CDK7 inhibitor QS1189 as an attractive option for efficiently suppressing MCL and other lymphoma cells. Additionally, QS1189 may be applicable for treating acquired resistance to venetoclax. Although the mechanisms of the anticancer effects of QS1189 included induction of G2/M arrest and apoptosis, further studies are needed before this QS1189 can be applied as an anticancer agent.

## Methods

### Cell culture and reagents

The human lymphoma cell lines (Mino, Maver-1, Jeko-1, Z-138, OCI-Ly7, OCI-Ly10, SU-DHL-6, SU-DHL-8, U2932, Daudi, Namalwa, Raji, and Ramos) were purchased from the American Type Culture Collection (ATCC, Manassas, VA, USA) and DSMZ (Braunschweig, Germany). Cells were cultured in RPMI1640 medium containing 10% foetal bovine serum, 100 U/mL penicillin, and 100 mg/mL streptomycin (Invitrogen, Carlsbad, CA, USA) at 37 °C in an atmosphere of 5% CO_2_. OCI-Ly7 and OCI-Ly10 were cultured in IMDM. Tests for mycoplasma contamination were negative. Ibrutinib and venetocalx were purchased from Selleck Chemicals (Houston, TX, USA).

### Generation of venetoclax-resistant cells

Maver-1/VR cells were established by chronic, repeated exposure to increasing venetoclax doses up to 1 μM for more than 8 months. Maver-1/VR cells were cloned by single-cell culture, and these resistant sublines were designated as Maver-1/VR-1 and Maver-VR-2. In all studies, resistant cells were cultured in drug-fee medium for >1 week to eliminate the effects of venetoclax. The genetic identity of each subclone with the parental cells was verified by short tandem repeat (STR) analysis conducted at Cosmogenetech (Cosmogenetech co. Ltd., Seoul, Korea) according to established protocols.

### MTT assay

Cells (2 × 10^4^) were seeded into 96-well sterile plastic plates overnight and then treated with the relevant drugs. After 72 h, 15 μL of MTT solution (5 mg/mL) was added to each well and the plates were incubated for 4 h. Crystalline formazan was solubilized with 100 μL of 10% (w/v) SDS solution for 24 h. Absorbance at 595 nm was read spectrophotometrically using a microplate reader. The results represent at least three independent experiments, and the error bars signify the standard deviation from the mean. The IC_50_ values were determined using GraphPad Prism software (GraphPad, Inc., La Jolla, CA, USA).

### Kinase profile assay

Kinase selectivity was assessed by the 33PanQinase® Activity Assay (ProQinase GmbH, Breisgau, Germany) consisting of 397 protein kinases and 13 lipid kinases for QS1189 at a single concentration (1 μM) using ATP Km for each kinase.

### Immunoblotting

Whole-cell lysates were prepared using EBC lysis buffer (50 mM Tris–HCl [pH 8.0], 120 mM NaCl, 1% Triton X-100, 1 mM EDTA, 1 mM EGTA, 0.3 mM phenylmethylsulfonylfluoride, 0.2 mM sodium orthovanadate, 0.5% NP-40, and 5 U/mL aprotinin) and then centrifuged. Proteins were separated by SDS-PAGE and transferred to polyvinylidene fluoride membranes (Invitrogen) for western blot analysis. The membranes were probed using antibodies against RNAPII CTD p-Ser2 (#13499), RNAPII CTD p-Ser5 (#13523), RNAPII CTD p-Ser7 (#13780), RNAPII CTD (#2629), aurora-A (#14475), aurora-B (#3094), PARP (#9541), caspase 3 (#9661), and Bim (#2819; all from Cell Signaling Technology, Danvers, MA, USA), CDK7 (sc-7344), cdc2 (sc-7344), cyclin B1 (sc-7393), cyclin D1 (sc-753), Bcl-xL (sc-1041), Bcl-2 (sc-7382), Mcl-1 (sc-819), Noxa (sc-56169), Puma (sc-374223), and β-actin (sc-47778; all from Santa Cruz Biotechnology, Dallas, TX, USA) as the primary antibody. The membranes were then treated with a horseradish peroxidase-conjugated secondary antibody. All membranes were developed using an enhanced chemiluminescence system (Thermo Scientific, Waltham, MA, USA).

### Lentiviral infection

Bcl-xL shRNA lentiviral vectors were purchased from Sigma-Aldrich (St. Louis, MO, USA). For lentiviral infection, cells were infected with shGFR or shBcl-xL (RNAi Consortium clone IDs; TRCN0000033503, #1; TRCN0000299585, #2) lentivirus. To evaluate cellular viability, cells were infected with shGFR or shBcl-xL for 72 h and then treated with 2 μg/mL puromycin for 48 h. The suppression of Bcl-xL was confirmed by immunoblotting before performing the MTT assay.

### RNA sequencing

Cells were treated with 1 μM QS1189 or vehicle for 6 h. Cells were pelleted, and total RNA was extracted using an RNeasy Mini Kit (Qiagen, Hilden, Germany). Genomic DNA was eliminated, and RNA integrity was verified using the Agilent 2100 Bioanalyzer (Agilent Technologies, Santa Clara, CA, USA). RNA Sequencing was performed by a specialized company (Macrogen, Seoul, Korea) using the TruSeq RNA Sample Prep Kit v2 and HiSeq. 2500 platform (Illumina, San Diego, CA, USA). Sequenced reads were trimmed using TrimMomatic 0.32. The trimmed reads were mapped to the hg19 reference genomes using HISAT (version 2.0.5) and Bowtie2. Expression levels were measured as kilobase of transcript per million mapped reads using StringTie (version 1.3.3b). A |log2 fold-change| ≥2.0 was set as the threshold to screen out differentially expressed genes. Gene-enrichment and functional annotation analysis and pathway analysis for significant gene lists were performed using the DAVID Bioinformatics Resources 6.8 (http://david.ncifcrf.gov).

### Flow cytometry analysis

Each cell was treated with the indicated doses of QS1189 for 24 h. To validate the cell cycle, cells were fixed in 70% ethanol at −20 °C from 1 h to a few days, incubated with 5 μL RNase (10 mg/mL), and finally stained with 10 μL propidium iodide (1 mg/mL). Cellular DNA content in the treated cells was analysed with a FACScan flow cytometer (BD Biosciences, Franklin Lakes, NJ, USA). Apoptosis was quantified using the annexin V-fluorescein isothiocyanate (FITC)/propidium iodide apoptosis kit (BD Biosciences) in accordance with the manufacturer’s protocols. Cells were resuspended in annexin V-binding buffer (150 mM NaCl, 18 mM CaCl_2_, 10 nM HEPES, 5 mM KCl, and 1 mM MgCl_2_). FITC-conjugated annexin V (1 μg/mL) and propidium iodide (50 μg/mL) were then added to the cells and incubated for 30 min at room temperature in the dark. Analyses were performed using an FACScan flow cytometer. Data were analysed with CellQuest software (BD Biosciences).

### Statistics

Data are presented as the mean ± standard deviation. *P* values were determined using unpaired or paired *t*-tests between groups using the GraphPad Prism software.

## Supplementary information

Table S1

Table S2

supplemtal information

## Data Availability

All data generated or analyzed during this study are included in this published article and the Supplementary Information.
