# Supplementary material for: Efficacy of the novel CDK7 inhibitor QS1189 in mantle cell lymphoma
Source: Sci Rep. 2019 May 10;9:7193. doi: 10.1038/s41598-019-43760-z (PMC6510728; doi:10.1038/s41598-019-43760-z)

Supplementary Information

Efficacy of the novel CDK7 inhibitor QS1189 in mantle cell lymphoma

Authors: Yun Jung Choi[1], Dong Ha Kim[1], Dok Hyun Yoon[2], Cheolwon Suh[2], Chang-Min Choi[2,3], Jae Cheol Lee[3], Jung Yong Hong[3,*], Jin Kyung Rho[4,*]

Affiliations:

[1]Asan Institute for Life Sciences, Asan Medical Center, University of Ulsan, College of Medicine, Seoul 05505, South Korea

[2]Department of Oncology, Asan Medical Center, University of Ulsan, College of Medicine, Seoul 05505, South Korea

[3]Department of Pulmonology and Critical Care Medicine, Asan Medical Center, University of Ulsan, College of Medicine, Seoul 05505, South Korea

[4]Department of Convergence Medicine, Asan Medical Center, University of Ulsan, College of Medicine, Seoul 05505, South Korea

*Corresponding authors:

Jung Yong Hong

Department of Oncology, University of Ulsan College of Medicine, 88, Olympic-ro 43-gil, Songpa-gu, Seoul 05505, South Korea. Tel: +82 2 3205 3208; Fax: +82 2 3010 6961; E-mail: hongjungyong@gmail.com

Jin Kyung Rho

Department of Convergence Medicine, University of Ulsan College of Medicine, 88,

Olympic-ro 43-gil, Songpa-gu, Seoul 05505, South Korea. Tel: +82 2 3010 2974; Fax: +82 2 3010 6961; E-mail: jkrho@amc.seoul.kr

Supplemental Methods

Cell-free kinase assay

A radioisotopic protein kinase assay (33PanQinase® Activity Assay, ProQinase GmbH, Freiburg, Germany) was used to measure the kinase activity of protein kinases including a panel of 28 CDK/cyclin complexes formed from 16 CDKs and 15 cyclins.

Supplemental table and figure legends

Table S1. List of targets profiled in Figure 1

Table S2. IC_{50} values of CDK/cyclin complexes by QS1189

Figure S1. Inhibition of RNAPII CTD phosphorylation by QS1189 treatment

MCL cells were treated with the indicated dose of QS1189 for 24 h. The indicated protein levels were analysed by immunoblotting.

Figure S2. GO analysis of G2/M arrest

RNA sequencing was performed as described in Figure 2. GO analysis of 3-fold upregulated or downregulated genes based on biological processes using DAVID software.

Figure S3. Modulation of anti-apoptotic protein by QS1189 treatment

Cells were treated with the indicated doses of QS1189 for 24 h. The indicated protein levels were analysed by immunoblotting.

Supplemental Figure S1

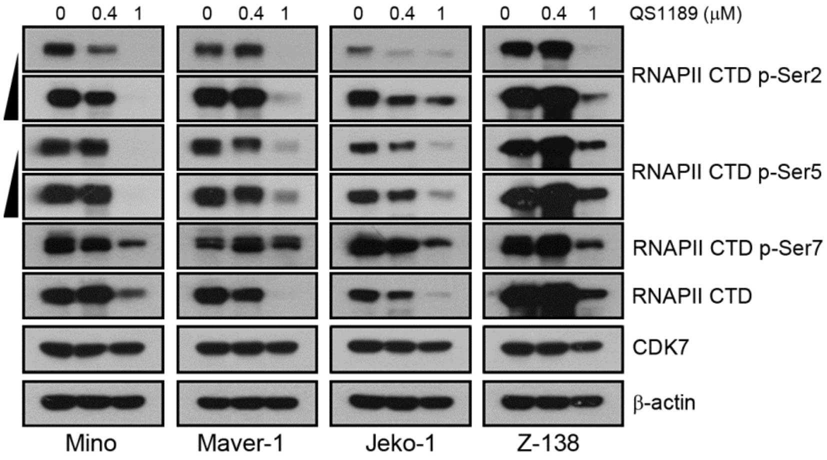

Supplemental Figure S2

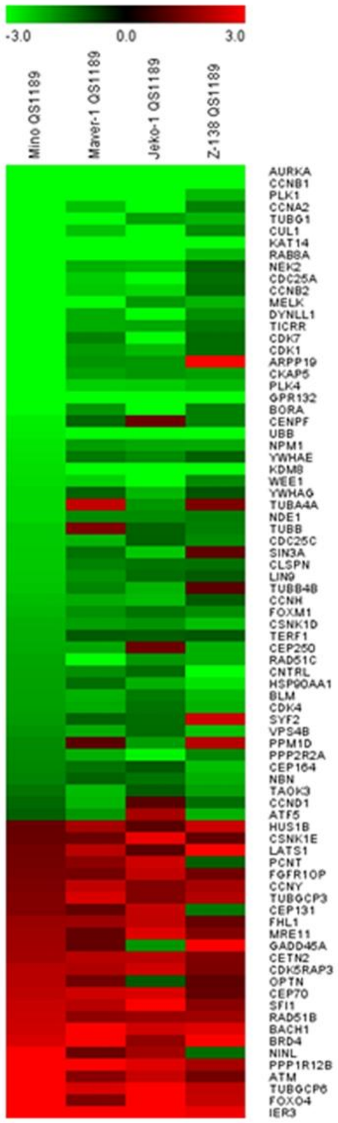

Supplemental Figure S3

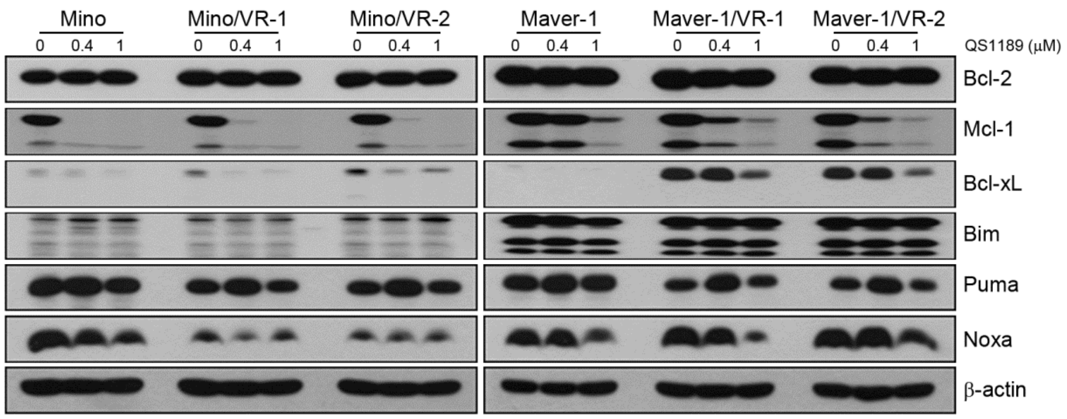

Full blots for figure 1c

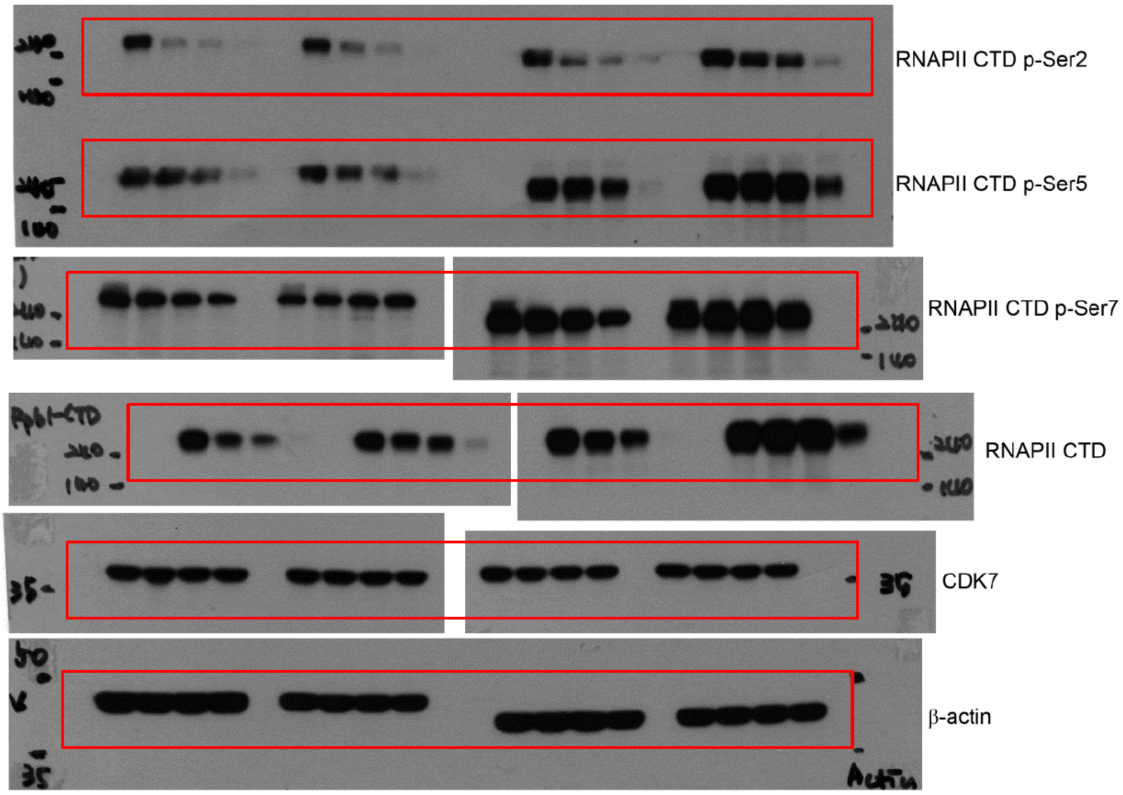

Full blots for figure 3b and d

3b

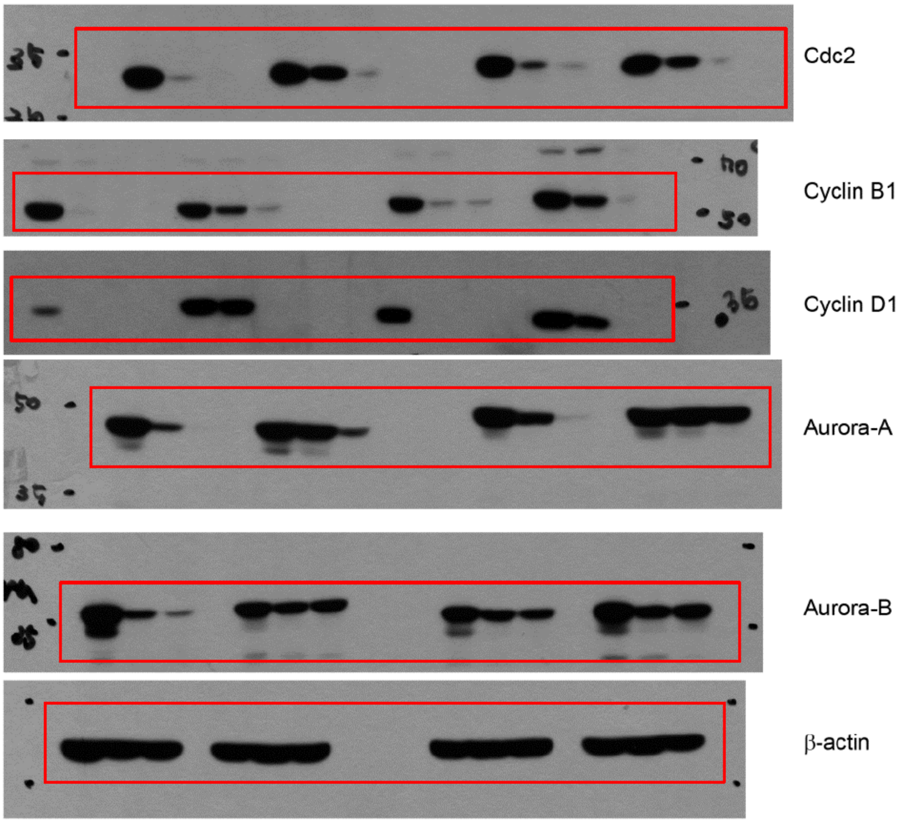

3d

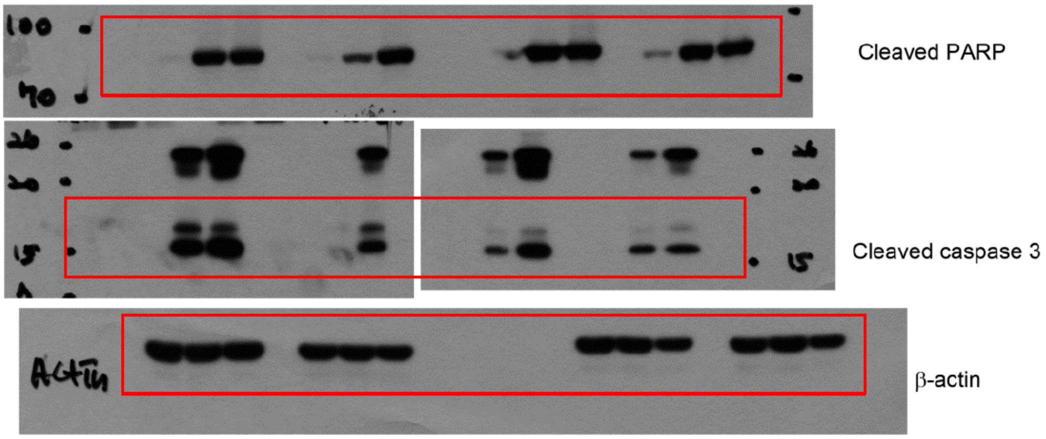

Full blots for figure 4b, c and f

4b

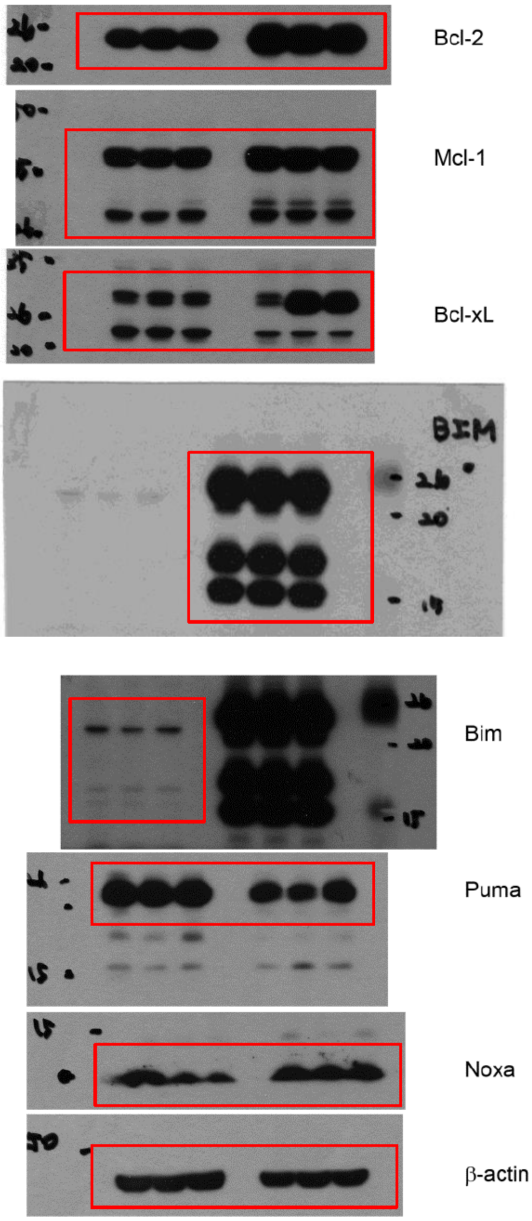

4c

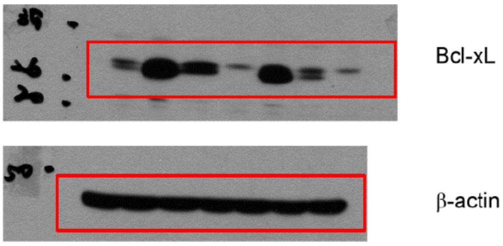

4f

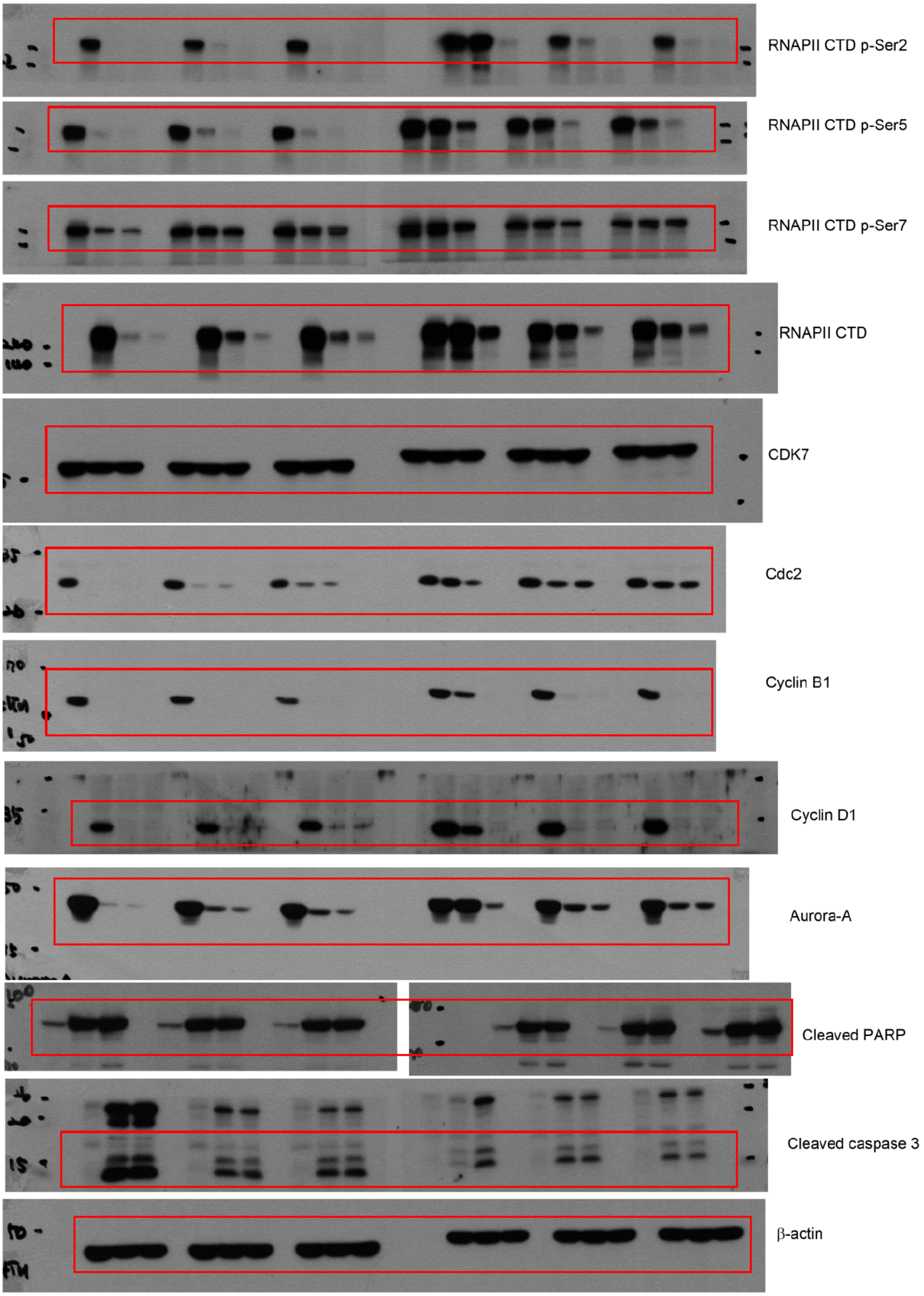
RNAPII CTD p-Ser2
RNAPII CTD p-Ser5
RNAPII CTD p-Ser7
RNAPII CTD
CDK7
Cdc2
Cyclin B1
Cyclin D1
Aurora-A
Cleaved PARP
Cleaved caspase 3
β-actin

Full blots for figure 5b

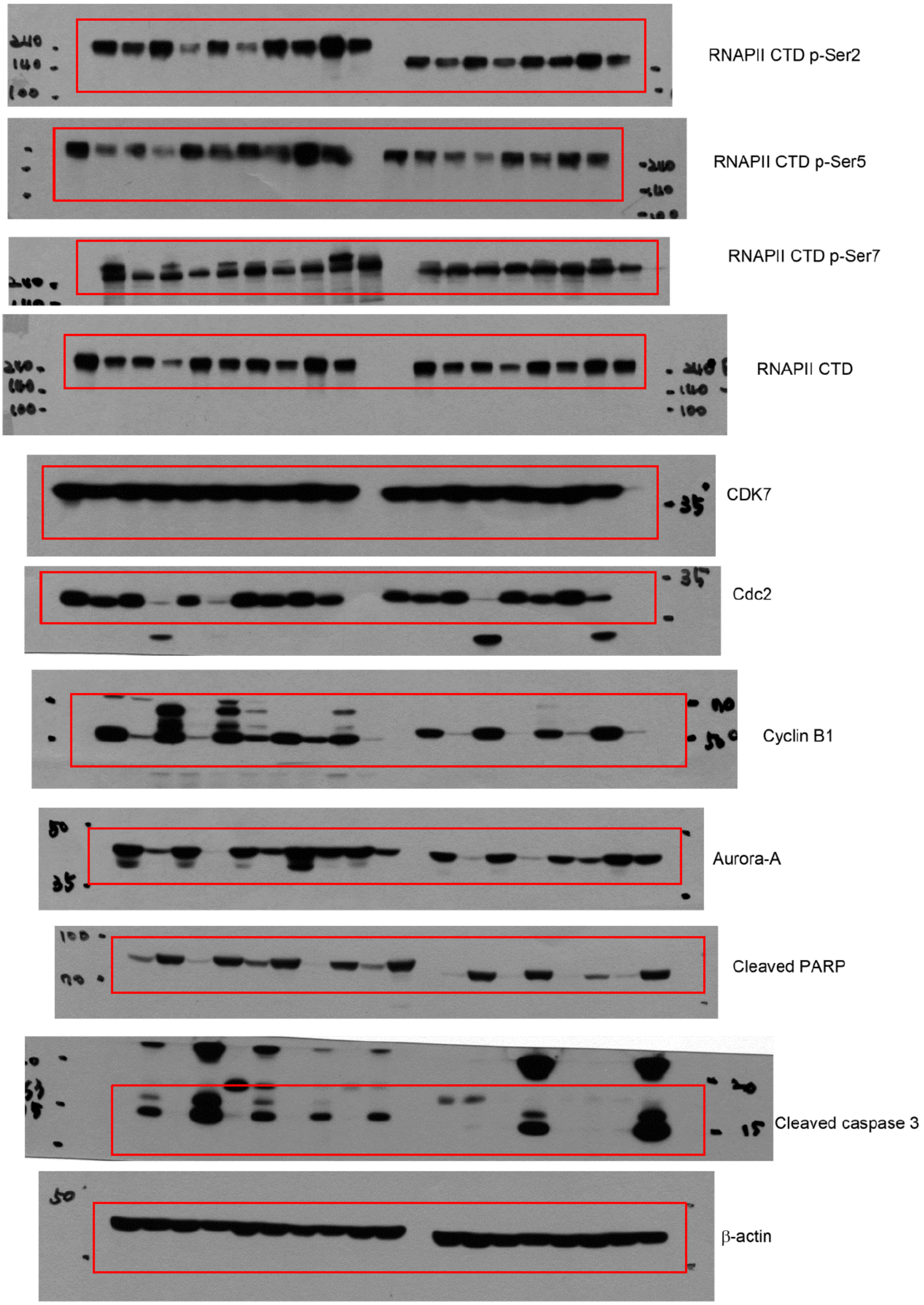

Full blots for supplemental figure S1

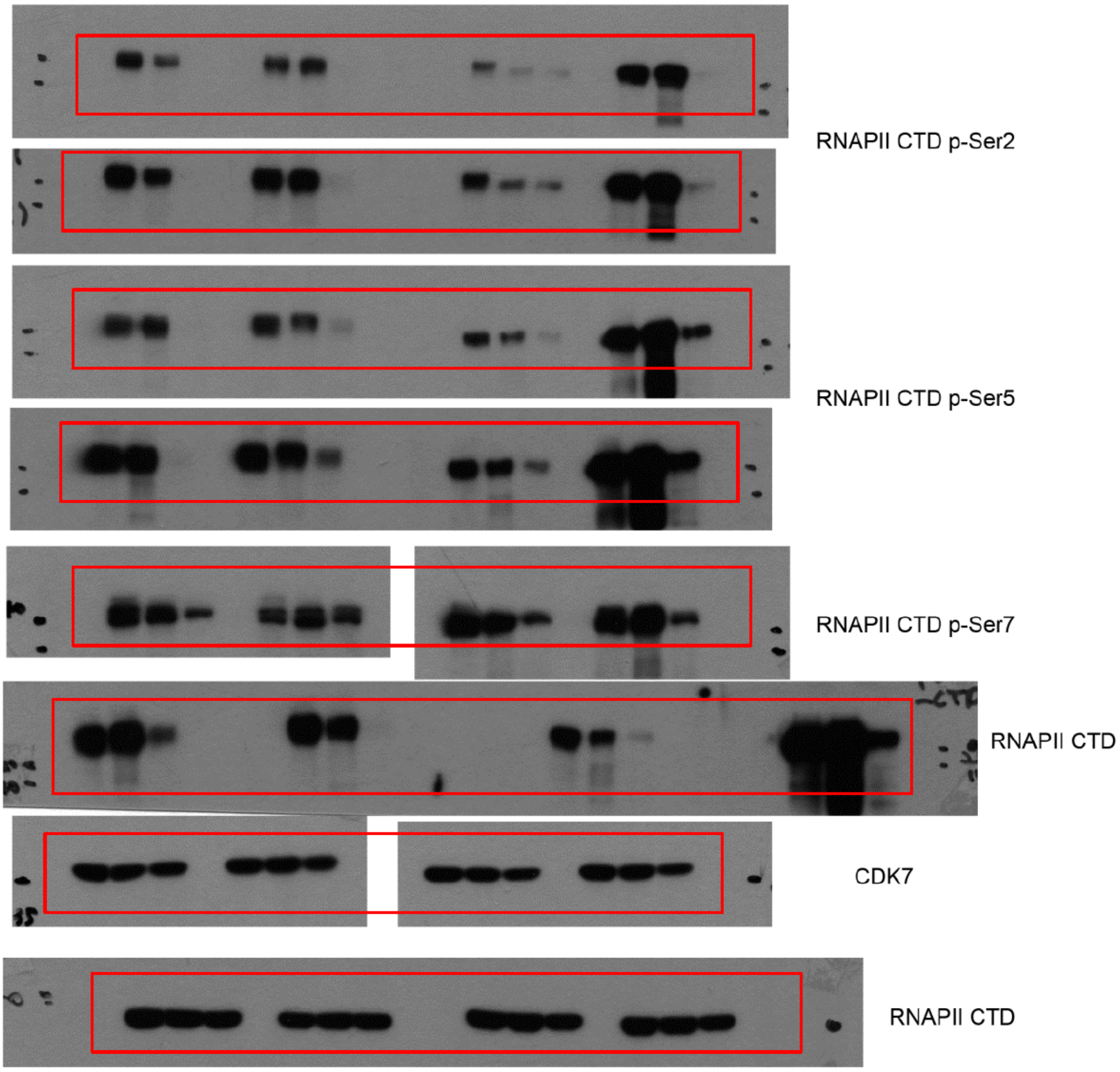

Full blots for supplemental figure S3

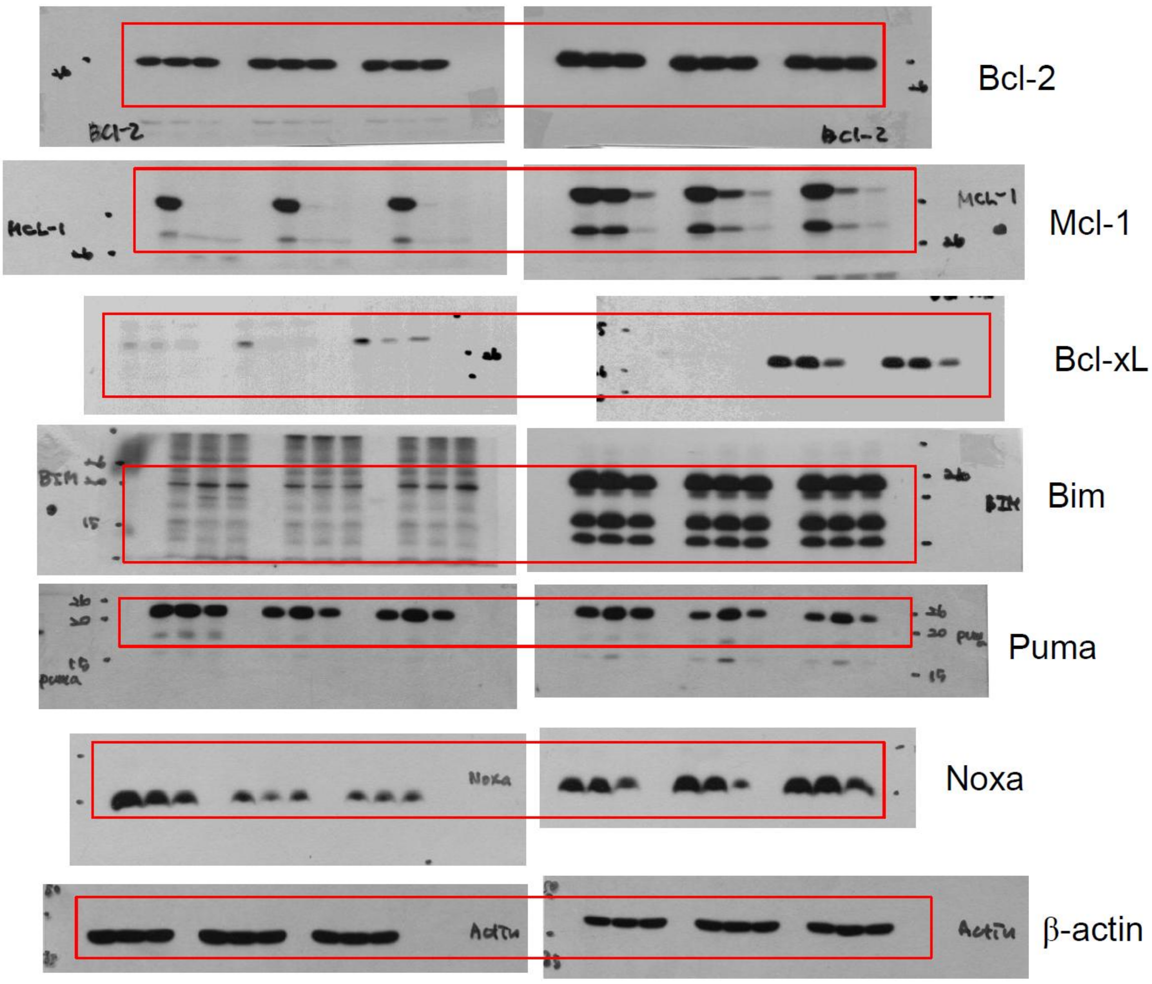

Supplement: Supplementary file 3 — supplemtal information [file 41598_2019_43760_MOESM3_ESM.pdf]
